# Single-Molecule Nanopore Sequencing of the TERT Core Promoter CpG Island Reveals PQS-Associated Strand Asymmetry of De Novo Methylation and Its Modulation by Photo-Oxidation

**DOI:** 10.3390/ijms27209129

**Published:** 2026-10-15

**Authors:** Alexander Sergeev, Adelya Genatullina, Viktoria Snyga, Mayya Monakhova, Elena Kubareva, Elizaveta Gromova, Maria Zvereva

**Affiliations:** 1 Department of Chemistry, M.V. Lomonosov Moscow State University, 119991 Moscow, Russiakubareva@belozersky.msu.ru (E.K.); gromova@belozersky.msu.ru (E.G.); zverevame@my.msu.ru (M.Z.); 2 Belozersky Research Institute of Physical and Chemical Biology, Moscow State University, 119991 Moscow, Russia

**Keywords:** *TERT* promoter, de novo DNA methylation, Dnmt3a, G-quadruplex, PQS, nanopore sequencing, oxidative DNA damage

## Abstract

DNA methylation of the human telomerase reverse transcriptase (*hTERT*) promoter contributes to telomerase regulation in cancer. We examined how strand identity and sequence context relate to methylation by the Dnmt3a catalytic domain (Dnmt3a-CD). Strand-resolved nanopore sequencing compared four promoter variants (wild type, C228T, C250T, and C228T/C250T) in amplicons and plasmids. Amplicons were also photo-oxidized with methylene blue before methylation. Methylation was higher on the cytosine-rich than on the guanine-rich strand. Defined putative quadruplex-forming sequence (PQS) cores showed reduced strand asymmetry and lower site-to-site variability; grouping sites by high G4Hunter scores did not reproduce this pattern. Variants and plasmid substrates largely retained the spatial profile. Photo-oxidized amplicons showed lower measured 5mC, particularly on the guanine-rich strand, while broadly retaining the profile. Photo-oxidation reduced sequencing quality. Bisulfite analysis of the C-rich strand of Raji DNA revealed a PQS-associated hypomethylation trough in both Dnmt3a-CD-treated DNA and an untreated aliquot. Together, these results highlight the importance of PQS sequence context for *hTERT* promoter methylation and the challenges of accurately sequencing guanine-rich, oxidation-prone DNA.

## 1. Introduction

The human telomerase reverse transcriptase gene (*hTERT*) encodes the catalytic subunit of telomerase and sets the replicative limit of the cell, so its reactivation is a near-universal step in cancer [1]. *hTERT* expression is controlled in part by DNA methylation of its promoter [2] and by recurrent core-promoter mutations, most often at the hotspot positions of somatic mutations C228T and C250T, also denoted −124C>T and −146C>T, respectively. These mutations were identified in several types of cancers as a key telomerase-activating event [3,4], and they create new binding motifs for ETS-family transcription factors [3,5], after which the GA-binding protein GABP is recruited to the mutant promoter and activates transcription [6]. Both altered methylation and the hotspot mutations are tightly associated with telomerase reactivation and cellular immortality [7].

Genetic and epigenetic variation are complementary components of intratumor heterogeneity. Studies of glioma progression and leukemia have linked variation in DNA methylation to tumor evolution, transcriptional diversity, and clinical outcome [8,9,10]. At TERT, promoter mutations are associated with allele-specific epigenetic states: expressed mutant alleles can be hypomethylated relative to transcriptionally silent alleles, and the relationship between methylation and expression depends on the promoter region examined [11,12]. These findings motivate testing whether the C228T and C250T sequence changes themselves alter susceptibility to de novo methylation in defined substrates.

The *hTERT* core promoter is unusually guanine-rich. It contains several sequences that can fold into G-quadruplexes (G4) under defined conditions, here called putative quadruplex-forming sequences (PQS). An extended 68-nt region contains three PQS and carries the C228T and C250T mutations; previous studies of synthetic hTERT fragments characterized G4 folding in this region [13].

Oxidative DNA damage adds a further dimension to the relationship between sequence context and methylation. Methylene-blue photosensitization of duplex DNA generates guanine lesions, with 8-oxo-7,8-dihydroguanine (8-oxoG) expected to predominate [14,15]. The treatment can also generate other products [16], providing a model for examining methylation after oxidative DNA damage.

De novo *TERT* promoter methylation rises during differentiation, more under atmospheric than hypoxic oxygen [17]. De novo methylation is performed by the DNMT3 family, which includes DNMT3A and DNMT3B [18]. Dnmt3a catalytic domains bind DNA cooperatively and can assemble into multimeric nucleoprotein complexes, while structural studies of the DNMT3A–DNMT3L complex revealed a tetramer containing two DNMT3A active sites [19,20]. Dnmt3a methylates duplex DNA with a strong preference for the sequence flanking the target CpG [21], while the PWWP domain of intact DNMT3A contributes to chromatin targeting and its ADD domain regulates access of DNA to the catalytic domain [22,23]. Two recent studies examined methylation of G4-containing promoter substrates: at the *MGMT* promoter, the guanine-rich strands of PQS were hypomethylated while the complementary cytosine-rich strands were methylated more efficiently [24], and at the *c-MYC* promoter, G4 trapped Dnmt3a-CD and blocked methylation locally [25]. Mouse Dnmt3a and human DNMT3A share 98% amino-acid identity, with highly conserved catalytic domains [26], supporting the use of mouse Dnmt3a-CD as a model for the intrinsic catalytic and flanking-sequence preferences of human DNMT3A. Here, Dnmt3a-CD is used to examine substrate-dependent methylation under defined in vitro conditions, without the PWWP and ADD regulatory domains.

How strand identity, defined PQS regions, and oxidation relate to de novo methylation at *hTERT* remains open. Here, single-molecule nanopore sequencing, which reads base modifications directly from the ionic-current signal without bisulfite conversion [27], was used to profile in vitro Dnmt3a-CD methylation of the *hTERT* core promoter (wild type and the clinically relevant C228T, C250T, and C228T/C250T variants), as PCR amplicons and as plasmid inserts. Amplicons were also examined after methylene-blue photo-oxidation. Four questions are addressed: (i) whether methylation depends on PQS proximity; (ii) how oxidation changes the methylation profile relative to PQS; (iii) whether the hotspot mutations alter it; and (iv) how methylation of human genomic DNA (as assessed by bisulfite sequencing) is distributed relative to PQS, compared to in vitro methylated substrates.

## 2. Results

### 2.1. Substrate Design and PQS Annotation of the hTERT Core Promoter

**Figure 1:**
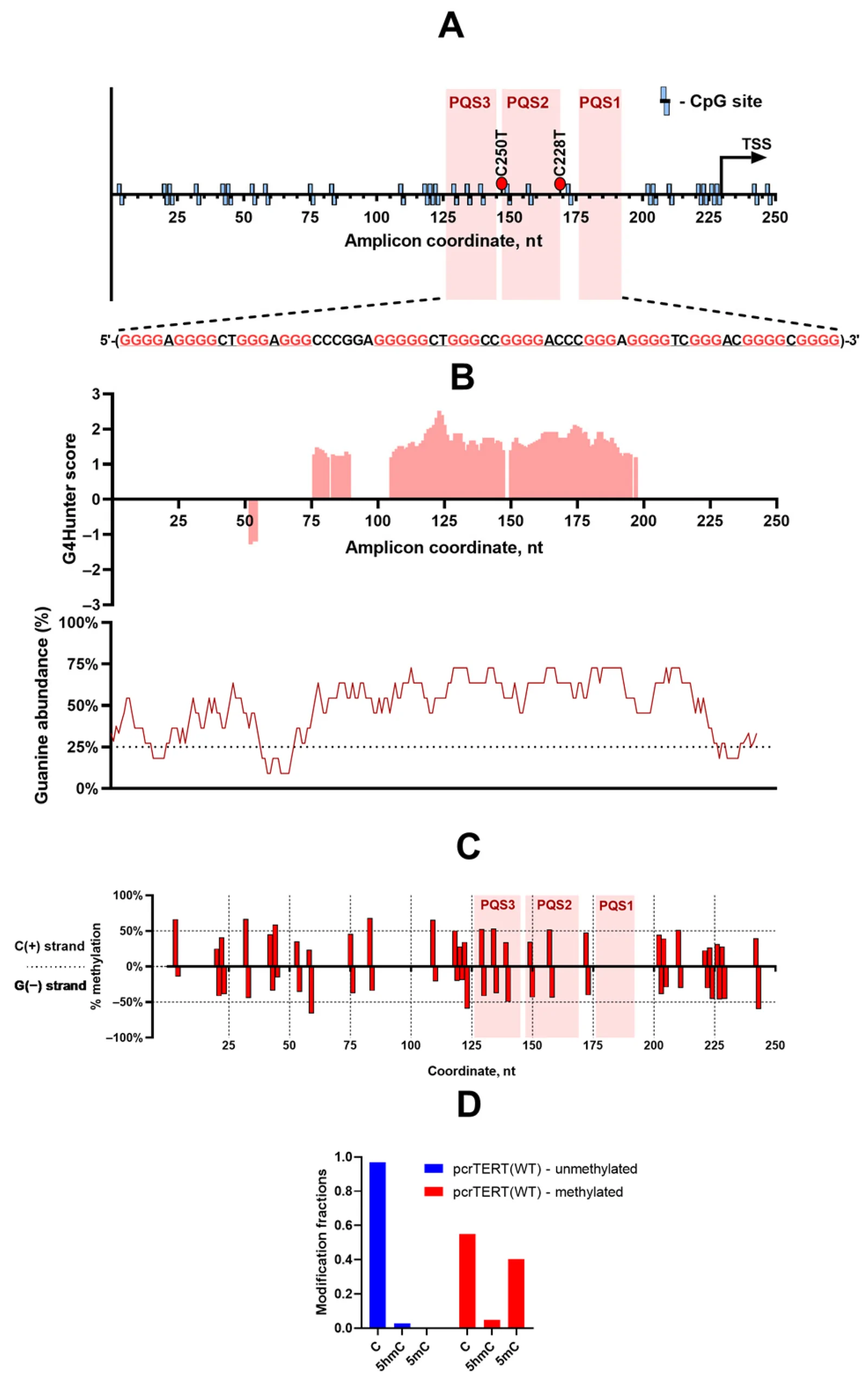
Substrate design and baseline de novo methylation of the wild-type *hTERT* core promoter. (**A**) Map of the *hTERT* core promoter amplicon in the shared coordinate frame. Tracks show the 27 promoter-insert CpGs and flanking reference positions, the three PQS (PQS3, 126–145; PQS2, 147–169; PQS1, 176–192) on both strands, the C250T (147) and C228T (169) hotspot positions within PQS2, and the transcription start site (229). The sequence of the PQS-containing region is expanded, and guanine residues assigned to G4 tetrads in the previously characterized oligonucleotide model [13] are highlighted in red. CpG sites are shown in light blue. (**B**) G4Hunter score along the amplicon for each strand (windowed; more negative on the C(+) strand indicates a G-richer, more G4-prone complementary strand) and a sliding G content (%G) track for the G(−) strand. The shaded 68-nt region carries the two hotspot mutations. (**C**) Single-CpG 5mC (5-methylcytosine) levels of the C(+) strand and the G(−) strand of wild-type pcrTERT(WT) after Dnmt3a-CD methylation. The PQS regions are shaded. Strand-specific methylation levels are shown as red bars. (**D**) Fractions of unmodified C, 5mC, and 5hmC calls in the WT control and Dnmt3a-CD-treated sample. One reaction was performed per condition.

To explore single-molecule nanopore sequencing as a tool for analyzing *hTERT* promoter methylation, PCR amplicons spanning the wild-type *hTERT* core promoter, pcrTERT(WT), the clinically relevant variants pcrTERT(C228T) and pcrTERT(C250T), and the double mutant pcrTERT(C228T/C250T) were amplified (Figure 1A) and methylated in vitro by the de novo methyltransferase Dnmt3a-CD. Sequencing yielded 306 Mb of amplicon data (Table S1). To place all observations in a single coordinate frame, the 27 CpGs within the promoter insert were numbered CpG1–CpG27 (Figure S1; Table S4), and the PQS cores and mutation sites were mapped to amplicon coordinates (PQS3, 126–145; PQS2, 147–169; PQS1, 176–192; C250T at 147 and C228T at 169, both within PQS2) (Figure 1A). Hereafter, ‘PQS cores’ refers specifically to these three coordinate intervals; broader G-rich or G4Hunter-positive regions are identified separately. The amplicon maps to the *hTERT* core promoter on the minus strand of chromosome 5 (GRCh38, NC_000005.10). The analyzed region spans chr5:1,295,036–1,295,270, and zero-based amplicon coordinate X corresponds to genomic position 1,295,282–X.

PQS annotation was based on sequence prediction and previous structural data. G4Hunter provides sequence-based quadruplex-propensity scores [28,29]; the defined PQS intervals were mapped to the shared coordinates shown in Figure 1A. Chemical probing, circular dichroism, and melting-temperature analysis have shown that synthetic 96-nt DNAs spanning this region fold into three stacked parallel G4s, and that every single mutation independently destabilizes the central quadruplex [13]. These earlier findings provide context for the sequence annotations. G4 folding during the present methylation reactions was not directly measured.

### 2.2. Baseline Modification Calling and De Novo Methylation of the Wild-Type Promoter

Analysis of unmethylated WT *hTERT* amplicons showed that the standard nanopore decoding model predicts false-positive 5-hydroxymethylcytosine (5hmC) and 5-methylcytosine (5mC) calls. Reliably distinguishing 5mC from 5hmC is a known challenge for current detection methods, including sequencing-based approaches [30]. Both a fixed 0.5 modification-quality threshold and modkit automatic filtering reduced these false-positive calls, but neither removed them. High-confidence 5hmC calls persisted at several discrete positions in the G/C-rich PQS region, most prominently on the G(−) strand, whereas residual false 5mC was substantially lower (Table S2). A 0.5 threshold was applied for downstream analyses. After filtering, the weighted false-positive 5mC rates in non-oxidized WT controls were 0.18% on C(+) and 0.31% on G(−); the corresponding 5hmC rates were 0.32% and 2.12% (Table S2). Figure 1D shows the overall modification-call composition of the control and treated WT samples.

Methylation differed between strands, reaching 36.0% on the C(+) strand and 31.4% on the G(−) strand (Figure 1C), showing different measured methylation on the two strands of the same fragment. Across all eight methylated amplicon samples, weighted C(+) strand methylation was higher than G(−) strand methylation. Among non-oxidized methylated amplicons, mean weighted methylation was 35.4 ± 3.8% (C) versus 28.6 ± 3.9% (G) (mean ± SD, across four variants), with a mean strand methylation ratio (C(+) strand over G(−) strand) of 1.24. The distinct C- and G(−) strand methylation profiles (Figure 1C) further show the positional strand difference. De novo methylation of the *hTERT* promoter by Dnmt3a-CD was therefore strand-asymmetric in the analyzed substrates, favoring C(+).

The increase in 5mC calls after Dnmt3a-CD treatment supports a methylation-associated signal. The 0.5 modification-probability filter reduced false-positive calls, and the remaining false-positive 5mC background in unmethylated controls was small compared with the measured strand difference. The principal profile was also retained in the plasmid pUC19(hTERT) substrate, which has different fragment ends (Section 2.3). Together, these controls argue against a simple background-calling explanation for the observed C > G asymmetry.

### 2.3. Reduced Strand Asymmetry and Site-to-Site Variability Within Defined PQS Cores

**Figure 2:**
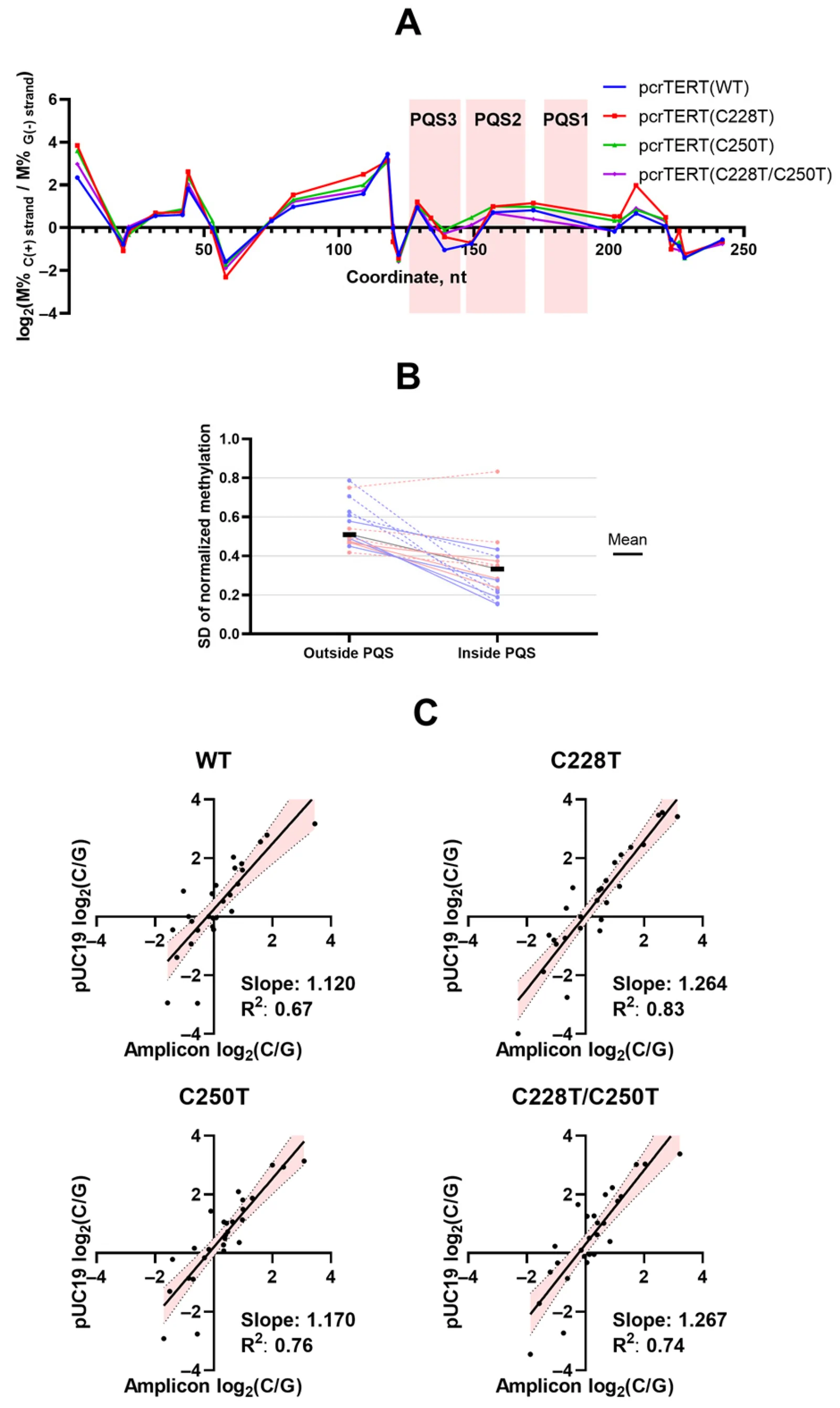
Asymmetric methylation, its equalization within PQS, and robustness to the hotspot mutations. (**A**) Per-CpG log_2_ strand methylation ratio, log_2_(C(+) strand/G(−) strand), along the amplicon for the four non-oxidized templates (wild type, C228T, C250T, C228T/C250T) overlaid on a common axis, with the PQS region shaded and a reference line at log_2_ = 0 (equal methylation). (**B**) Within-sample variability of normalized methylation, plotted as one connecting line per template × oxidation-state × strand group (16 groups) between flanking sequence and PQS cores. Group means are shown as black bars. (**C**) Methylation asymmetry (log_2_(C(+) strand 5mC/ G(−) strand 5mC)) on the pUC19(hTERT) insert versus the amplicon. Solid lines show linear regressions, shaded areas show 95% confidence intervals, and the slope and R^2^ are reported in each panel. One reaction was performed per condition; the regression bands describe the site-level fit and do not estimate variation between independent reactions.

Next, the strand methylation ratio (C(+) strand over G(−) strand) was resolved by position along the amplicon for each template (Figure 2A). In the broader GC-rich region encompassing PQS1–3 (~98–194 nt of the amplicon), the strand methylation ratio was close to 1, whereas in the flanking sequence the two strands were unequally methylated, with the ratio ranging from ~5 to ~0.3. Thus, measured methylation in the PQS-containing region was more even across strands than in the surrounding sequence. Along the sequence, the C- and G(−) strand profiles oscillated, starting at the PQS1–3 region (Figure 1C).

This strand equalization reflects a broader smoothing effect specific to defined PQS cores rather than to general G-richness. Within PQS cores, the methylation profile was smoother, with a lower standard deviation of normalized methylation per sample and strand than outside (median within-sample SD, 0.308 vs. 0.511), a direction observed in 15 of 16 template × oxidation-state × strand groups (Figure 2B). By contrast, grouping positions by a high G4Hunter score, rather than by the defined cores, gave almost no smoothing (median SD, 0.513 vs. 0.488; lower variability in 8 of 16 groups; Table S4). The strand methylation ratio was organized into distinct segments rather than a single gradient: a variable, high-ratio transition around 106–125, a smoother PQS core (126–170), and a terminal segment beyond ~226 nt where the G(−) strand became more methylated. Consistent with the absence of a simple gradient, the position-by-position correlation of methylation with amplicon coordinate was weak (Pearson r = −0.075), as was that with the G4Hunter score (r = 0.053). Within the 27-CpG WT promoter insert, the unweighted mean C-strand levels were similar inside PQS cores and in the flanks (33.7% and 35.3%).

Adding the C228T, C250T, or C228T/C250T mutations had only a minor effect on the strand methylation ratio: the profile was preserved across all variants, with no substantial change detected under the tested conditions (pairwise profile correlation r = 0.94–0.98; Figure 2A). Aggregate template-corrected context profiles of the four templates were highly similar (Pearson r = 0.89–0.97). The de novo pattern written by Dnmt3a in this promoter was therefore largely retained in the sequence variants, indicating that the mutations alone did not substantially alter the Dnmt3a-CD methylation profile in this purified in vitro system.

To examine methylation in a substrate without the physical ends of the short linear amplicon, the analysis was repeated on pUC19 plasmids carrying a 235-bp insert corresponding to the *hTERT* promoter fragment pUC19(hTERT) with the four variants—WT, C228T, C250T and C228T/C250T (Figure 2C). The plasmid results largely retained the amplicon methylation pattern (site-level 5mC Pearson r = 0.820; strand-ratio r = 0.872) and showed an even stronger C > G bias. Thus, the principal positional pattern was retained in a longer circular DNA context with vector-derived flanks and was not restricted to the short amplicon substrate.

### 2.4. Lower Measured Methylation After Dnmt3a-CD Treatment of Photo-Oxidized DNA

**Figure 3:**
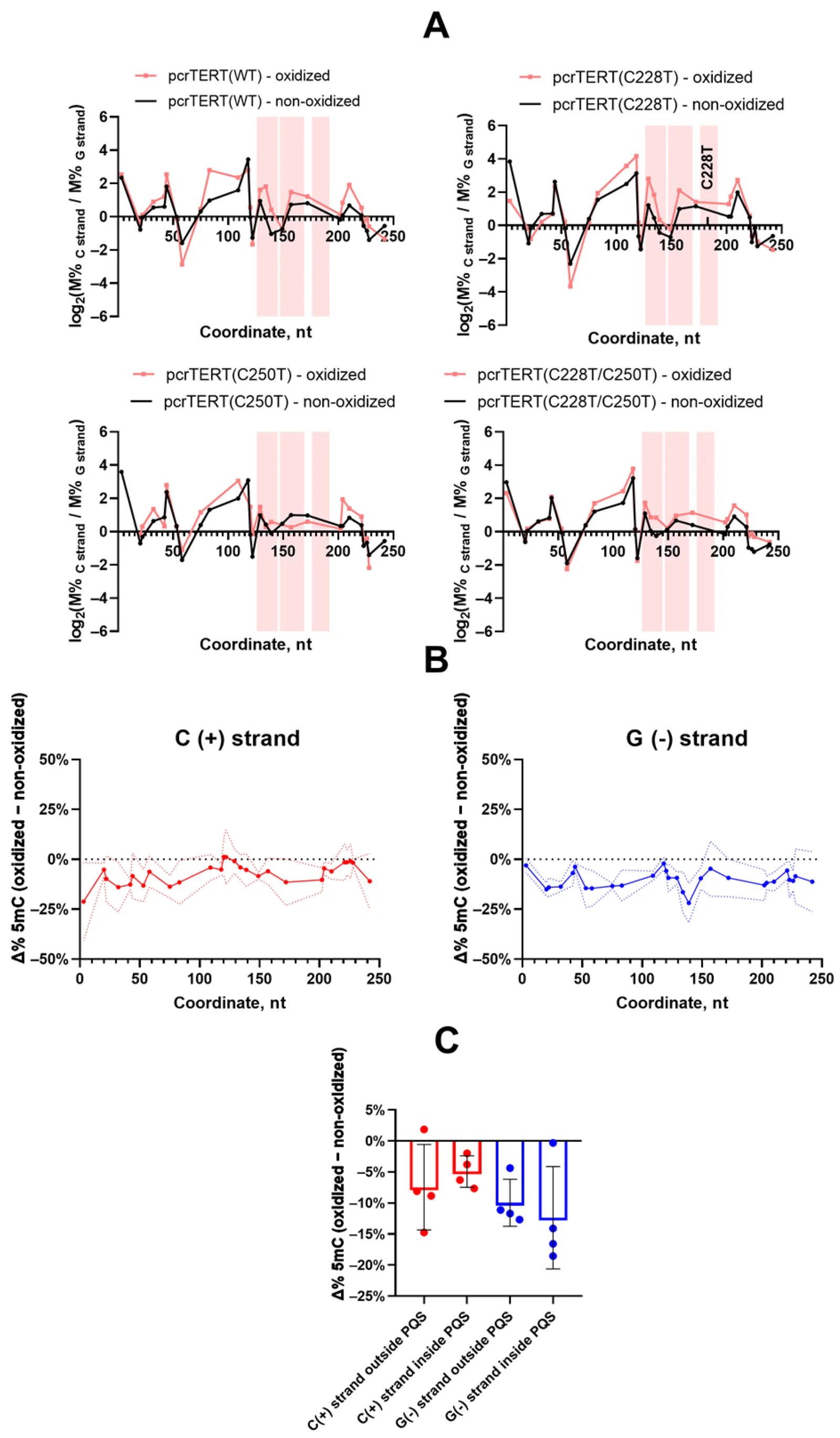
Lower measured methylation after Dnmt3a-CD treatment of photo-oxidized DNA, with a larger decrease on G(−). (**A**) Per-CpG log_2_ strand methylation ratio for oxidized DNA (reference line at 0, PQS shaded), with the non-oxidized profile overlaid in black. (**B**) Per-CpG change in methylation on oxidation (oxidized—non-oxidized), plotted separately for the C (left panel) and G (right panel) strands as the mean ± SD across the four promoter variants. (**C**) Oxidation-induced methylation change inside versus outside the PQS cores, by strand (bars = mean over the four variants; error bars = SD; points = individual variants). One reaction was performed per condition.

To probe how oxidative DNA damage affects de novo methylation, amplicons were first oxidized with methylene blue under visible light [14] and then methylated by Dnmt3a-CD. Comparison of separated strands showed that photo-oxidation increased the mean C/G methylation ratio (Figure 3A). As for non-oxidized fragments, the PQS region (~98–194 nt) retained a strand methylation ratio near 1, while the flanking sequence remained unequal over the same range. Overall, oxidation was associated with lower measured methylation in the Dnmt3a-CD-treated amplicons (weighted 32.0 ± 3.7% → 23.8 ± 7.9%, mean ± SD across four variants after averaging the two strand-weighted percentages for each variant). Both strands showed lower methylation, the G(−) strand more strongly (oxidized means 28.8% C vs. 18.9% G; mean variant-specific C/G ratio 1.57; non-oxidized ratio 1.24). The change was mainly in the overall level of measured methylation, while its distribution along the amplicon was broadly retained: matched oxidized and non-oxidized profiles remained strongly correlated along the amplicon (mean Pearson r = 0.87), and the aggregate sequence-context profiles were also similar (r = 0.948).

As under non-oxidized conditions, the oxidized strand-ratio profiles of the four templates were broadly similar, so the main profile was retained across variants after oxidation (Figure 3A). One local difference emerged within this preserved pattern: as an exploratory observation, the single mutations C228T or C250T, but not the double variant, appeared to raise the strand methylation ratio at CpG10 (coordinate 109) under oxidation, while the rest of the profile was preserved (Figure 3A). The decrease in measured methylation was strand-dependent and greater on the G(−) strand across the four template pairs (Figure 3B). The decrease was more strongly guanine-strand-specific within the PQS cores (G −12.4 vs. C −4.9 percentage points) than in flanking sequence (G −10.0 vs. C −7.5) (Figure 3C). Photo-oxidation was therefore associated with a strand-dependent decrease in measured methylation, with a larger mean G-strand change in the PQS cores than in the flanks (Figure 3B,C).

**Figure 4:**
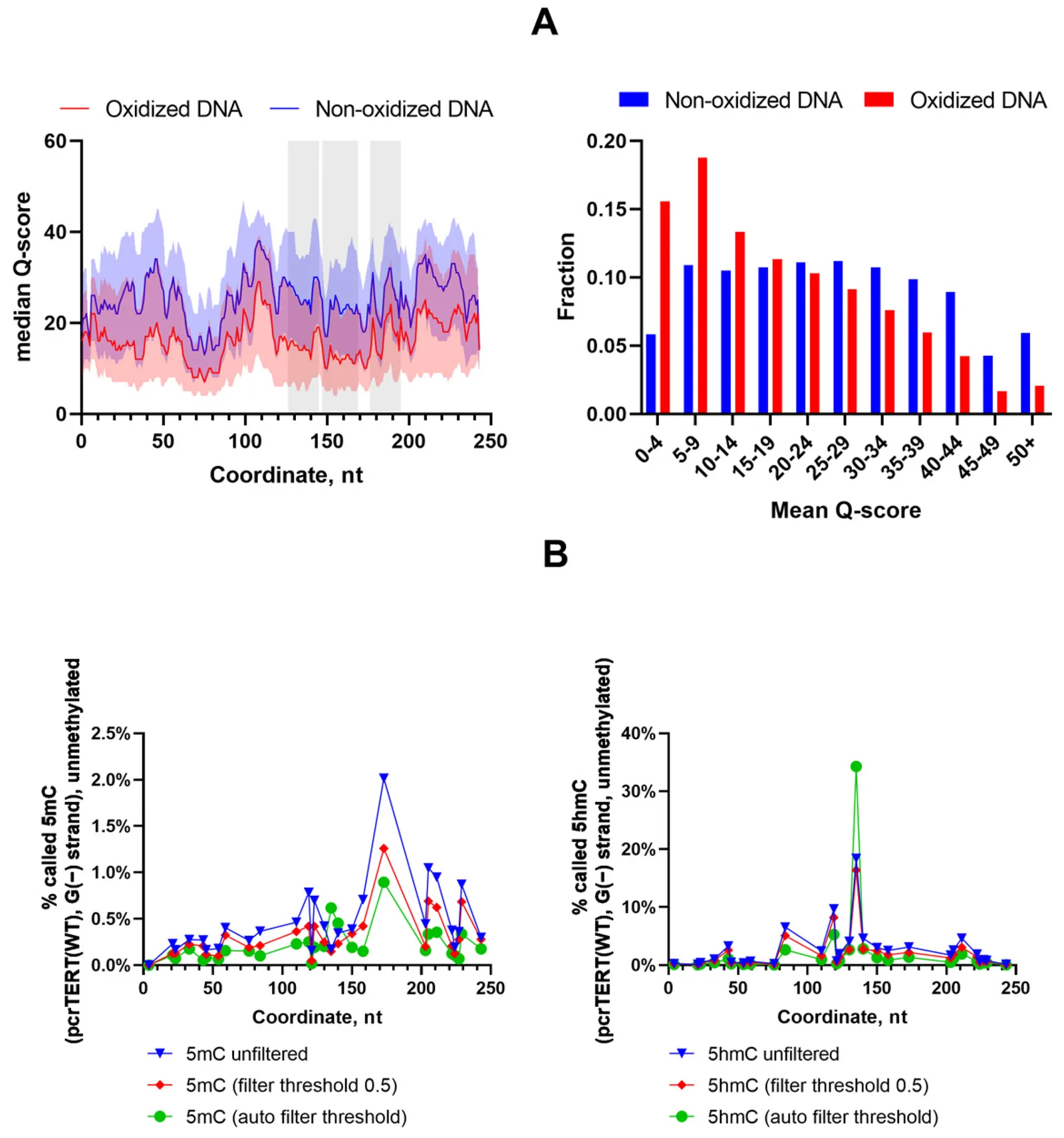
Reliability of single-molecule reading in guanine-rich, oxidation-prone DNA. (**A**) Coordinate-resolved median base quality along the amplicon and the pooled distribution of individual base-quality values for non-oxidized and oxidized DNA, with the interquartile band shaded and PQS regions marked. (**B**) Position-resolved false-positive modification calls on the G(−) strand of unmethylated control amplicons (non-oxidized) under three filtering modes (no explicit threshold, fixed 0.5 threshold, modkit automatic filtering): 5mC (left) and 5hmC (right), PQS shaded. Filtering reduces the calls but does not remove a high-confidence 5hmC hotspot at coordinate 135 (PQS3), whereas residual 5mC stays low. Full conditions (C(+) strand, oxidized DNA, aggregate rates) are in Table S2. One reaction was performed per condition.

Oxidation also reduced sequencing quality (Table S1). Photo-oxidation lowered base quality across the whole amplicon rather than at isolated points (Figure 4A): mean base quality fell from Q25.2 to Q18.0, and the fraction of low-quality bases (Q < 10) rose from 16.7% to 34.3%, with the lowest-quality stretches around coordinates 64–85 and PQS2. Both stretches fall in guanine-rich segments (G4Hunter score ≈ −1.2 to −2.1), so the lowest-quality stretches coincided with G-rich segments (Figure 4A). False-positive modification calls in unmethylated controls were examined under three filtering modes. Figure 4B shows the non-oxidized G(−)-strand control; Table S2 reports the other conditions, including photo-oxidized controls. Filtering reduced these calls but did not remove a high-confidence 5hmC hotspot at coordinate 135 within PQS3, whereas residual 5mC remained low.

### 2.5. Bisulfite Sequencing Reveals a PQS-Associated Hypomethylation Trough in Raji DNA

**Figure 5:**
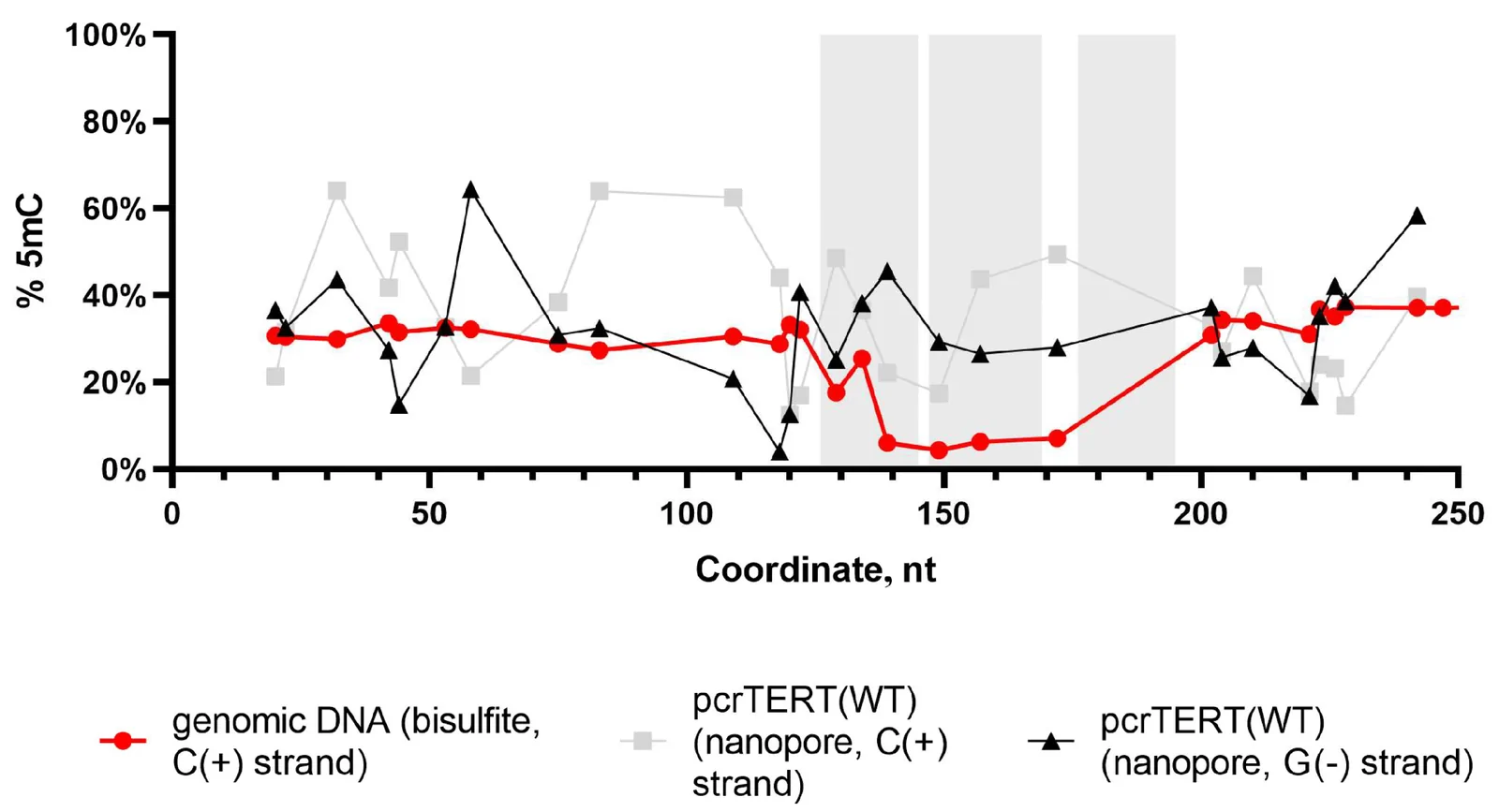
Bisulfite sequencing of Dnmt3a-CD-treated Raji DNA reveals a PQS-associated hypomethylation trough. Per-CpG bisulfite methylation of Dnmt3a-CD-treated Raji cell line genomic DNA along the amplicon coordinate frame (PQS shaded), overlaid with the matched pcrTERT(WT) nanopore C(+) strand and, faintly, the nanopore G(−) strand. The bisulfite amplicon reads the C(+) strand. One reaction was performed per condition.

To examine methylation in a longer genomic DNA context, genomic DNA from Raji cells, a human Burkitt lymphoma B-cell line, was treated with Dnmt3a-CD and profiled by bisulfite sequencing across the *hTERT* promoter (Figure 5, Table S3).

The bisulfite profile contained a sharp hypomethylation trough at CpG16–19 (amplicon coordinates 139–172), where methylation estimates were 6.08%, 4.36%, 6.31%, and 7.12%, respectively, with shoulders at CpG14 (17.6%) and CpG15 (25.4%) and a sharp recovery beyond (Figure 5). Across the matched CpGs, methylation in the PQS3/PQS2 cores (coordinates 126–169) averaged 12.0%, against 31.2% in flanking sequence, a 2.6-fold difference. The flanking CpGs with the highest G4Hunter scores (coordinates 118–122) remained appreciably methylated (29–33%), whereas the trough coincided with the PQS cores in which the two nanopore strands were more evenly methylated and the profile was smoother (Figure 2B). Converted-T calls within PQS3/PQS2 had mean base qualities of approximately Q37–41 (Table S3).

An aliquot of the same starting DNA preparation analyzed without in vitro Dnmt3a-CD treatment also showed the central trough (Figure S2). Methylation estimates at CpG16–19 were 11.1%, 0%, 5.3%, and 12.5% under the reported analysis settings. The observed zero was restricted to CpG17. CpG16–18 lie within the annotated PQS3/PQS2 cores, whereas CpG19 lies in the adjacent gap.

The shared trough reflects endogenous *hTERT* promoter methylation in Raji DNA. No additional methylation attributable to Dnmt3a-CD was detected in this comparison. Control estimates on the flanks were higher than in the treated dataset under the main analysis settings. This trough was specific to the Raji preparation among the tested substrates: the amplicon C(+) strand was not suppressed in PQS (33.7% vs. 35.3% in flanking sequence), and neither was the plasmid pUC19(hTERT) (Section 2.3).

## 3. Discussion

The sequence specificity of de novo methylation by DNMT3A has been attributed primarily to the local context of the target CpG. Catalytic efficiency at a given site is governed largely by the immediately flanking nucleotides, a preference pronounced enough to shape the human epigenome [21]. The present analysis of the *hTERT* core promoter reveals a regional pattern in addition to these local preferences. The methylation pattern is associated with defined PQS regions, which extend well beyond the target CpG and its proximal flanks. Within these regions, strand asymmetry and site-to-site variability were lower, whereas methylation showed little correlation with G4Hunter score.

The de novo methylation pattern deposited by Dnmt3a-CD on the *hTERT* core promoter sequence is associated with the specific PQS for this region and is not captured by predicted G4-forming potential alone. It is strand-asymmetric outside PQS, more even across strands and smoother within PQS cores, reduced in magnitude but broadly retained after photo-oxidation, and largely unchanged by the clinical hotspot mutations. Raji DNA showed a distinct feature in the corresponding promoter region: a pronounced hypomethylation trough.

Defined PQS cores showed reduced strand asymmetry and site-to-site variability. PQS cores brought the strand methylation ratio toward unity and reduced within-strand variability, whereas a high G4Hunter score alone produced no comparable smoothing. The observed difference therefore did not scale with sequence-predicted G4 propensity. In the amplicon, the similar C-strand methylation levels within PQS and in the flanks (33.7% and 35.3%) distinguish this pattern from regional hypomethylation. Its retention in the plasmid shows that the principal profile is not confined to the short linear substrate. Earlier work at MGMT and c-MYC also identified methylation differences in G4-containing promoter substrates [24,25], providing a useful context for the sequence-associated profiles observed here.

In purified Raji genomic DNA, the C(+) strand of the PQS3/PQS2 region was deeply hypomethylated, whereas the methylase-treated short amplicon pcrTERT(WT) and the plasmid pUC19(*hTERT*) retained methylation in that region. This result identifies a feature of endogenous methylation, alongside the strand equalization and smoothing seen in model substrates.

Photo-oxidation of DNA substrates was associated with lower measured methylation without broadly reorganizing its spatial pattern, and the decrease was greater on the G(−) strand (matched site-level profile r = 0.87). Oxidative lesions could affect productive enzyme–DNA interactions. In lesion-defined duplexes, 8-oxoG altered Dnmt3a methylation in a position-dependent manner, with particularly strong inhibition when it replaced the guanine immediately following the target cytosine within CpG [31]. This provides a possible biochemical explanation for a lower methylation level with a retained positional profile. Methylene-blue photosensitization produces a mixture of guanine lesions [16], so the present result concerns oxidative damage as a whole. Photo-oxidation also lowered sequencing quality, and independent experiments show that 8-oxoG can perturb nanopore signals and standard basecalling [32]. Quality-dependent calling effects may therefore contribute to the measured decrease; unmethylated controls estimate false-positive background but do not calibrate false negatives in damaged, methylated DNA.

In cells, sequence preferences act together with chromatin regulation. Nucleosome contacts can position a DNMT3A2-containing complex toward linker DNA through a DNMT3B3 contact with the acidic patch [33], while reconstituted nucleosomes strongly protect core DNA from methylation [34]. Intact DNMT3A also undergoes PWWP-dependent recruitment to H3K36 methylation [22] and relief of ADD-mediated autoinhibition by H3 bearing unmethylated K4 [23]. These interactions regulate which CpGs are accessible to the enzyme. The catalytic domain assays isolate the substrate-dependent component, providing a basis for examining how this regional profile is modified in chromatin.

Two practical implications follow our results. First, strand-asymmetric methylation in the model substrates shows how an assay reading a single strand, or averaging the two strands, can obscure strand-specific states of the *hTERT* promoter. The strands inside the PQS region were methylated more evenly than in the flanks. The largely retained profile in the C228T/C250T variants indicates that these substitutions alone did not substantially alter Dnmt3a-CD methylation under the purified in vitro conditions. Their effects in chromatin may differ: allele-specific methylation and expression at *TERT* depend on the promoter region and cellular context [11,12]. Transcription-factor recruitment and chromatin regulation can connect promoter mutations with epigenetic differences, contributing to the genetic and epigenetic heterogeneity of tumors. Second, false-positive 5mC and 5hmC calls in G-rich, oxidation-prone sequences complicate single-molecule studies of this locus. A modification probability threshold reduces this background, making strand- and position-specific controls useful when interpreting these profiles.

## 4. Materials and Methods

### 4.1. DNA Fragments

A 256-bp DNA fragment containing the *hTERT* promoter region was obtained by PCR from human genomic DNA (Human Genomic DNA 11691112001, Roche, Boulogne-Billancourt, France). The primers used were: ATAGGTACCAGTGGATTCGCGGGCACAGA and AGTCAAAAGCTTCAGCGCTGCCTGAAACT. The fragment was inserted into the pUC19 vector at the KpnI and HindIII sites, following standard protocols. To introduce the C228T and C250T substitutions (historically denoted −124C>T and −146C>T, respectively), the QuikChange protocol was used (Stratagene, San Diego, CA, USA). The inserted sequences were verified by Sanger sequencing.

DNA fragments containing the *hTERT* promoter region and carrying the driver mutations at C228T, C250T, or both were obtained by PCR amplification from the plasmids pUC19(hTERT)-WT, pUC19(hTERT)-C228T, pUC19(hTERT)-C250T, and pUC19(hTERT)-C228T/C250T using Hot Start Taq polymerase and the primers: 5′-ATAGGTACCAGTGGATTCGCGGGCACAGA-3′ and 5′-AGTCAAAAGCTTCAGCGCTGCCTGAAACT-3′. This gave four 256-bp products: pcrTERT(WT), pcrTERT(C228T), pcrTERT(C250T), and pcrTERT(C228T/C250T). Plasmids were isolated from *E. coli* JM109 (dcm^−^) using a Plasmid Midiprep kit (Evrogen, Moscow, Russia).

### 4.2. Expression and Purification of Dnmt3a-CD

The catalytic domain of mouse DNA methyltransferase Dnmt3a (Dnmt3a-CD) bearing an N-terminal His_6_-tag was expressed in *E. coli* BL21(DE3) transformed with a pET-28a(+) construct [31]. Cells were lysed by sonication and the supernatant clarified by centrifugation. His_6_-Dnmt3a-CD was purified by metal-affinity chromatography on Co^2+^-charged TALON resin and eluted with a stepwise imidazole gradient. Purification was monitored by 12% SDS-PAGE. The Dnmt3a-CD concentration was 13 µM (0.5 mg of protein per litre of culture; ≥90% purity) as measured using Bradford reagent (Sigma, Burlington, VT, USA).

### 4.3. In Vitro Methylation

In vitro methylation by Dnmt3a-CD was performed on both pUC19-based plasmids carrying the *hTERT* insert and the *hTERT* promoter PCR products. DNA (1 µg) was incubated with 2 µM Dnmt3a-CD in reaction buffer (20 mM HEPES-NaOH pH 7.5, 100 mM KCl, 1 mM EDTA, 1 mM 1,4-dithiothreitol, 5% glycerol) with 25 µM AdoMet for 1 h at 37 °C. The enzyme was inactivated at 65 °C for 20 min, and methylated DNA was purified with the GentaPure Cleanup kit (Genterra, Moscow, Russia) per the manufacturer’s instructions.

### 4.4. DNA Oxidation

For oxidized-plus-methylated samples, PCR products were first subjected to methylene-blue-mediated photo-oxidation [14], purified, and subsequently methylated with Dnmt3a-CD. Matched photo-oxidized samples not treated with Dnmt3a-CD were used as oxidation controls. 2 µL of 0.1% methylene blue was added to 20 µL of DNA (30 ng/µL) in 10 mM Tris-HCl (pH 8.0), 1 mM EDTA, and the sample was irradiated on ice with a 35 W lamp placed 5 cm from the tubes for 15 min. Oxidized DNA was recovered by ethanol precipitation. The composition, abundance, and distribution of the resulting lesions were not measured.

### 4.5. Nanopore Sequencing and Base-Modification Calling

Samples were prepared for the MinION Mk1B sequencer (R10.4.1 flow cell) using the “Ligation sequencing amplicons—Native Barcoding Kit 24 V14 (SQK-NBD114.24)” protocol (Oxford Nanopore, Oxford, UK). Methylation status of the plasmids and PCR products was determined by comparison with matched unmethylated control DNA. A total of 306 Mb (amplicons) and 4.1 GB (plasmids) of data were obtained. Basecalling and modification calling used the model dna_r10.4.1_e8.2_400bps_sup@v5.2.0_5mCG_5hmCG@v2. Stranded per-CpG 5mC was obtained with modkit pileup--cpg. A base-modification quality filter threshold of 0.5 was applied to suppress false 5mC/5hmC calls. This value was chosen because 5mC and 5hmC predictions were essentially unchanged at thresholds of 0.5 and above, whereas higher thresholds reduced post-filter read depth without improving specificity. For the false-positive analysis, unmethylated control BAMs were reprocessed with modkit pileup--cpg preserving both 5mC and 5hmC, under three filtering modes (no explicit threshold, the fixed 0.5 threshold, and modkit automatic filtering). Position-resolved false-positive rates are weighted modification percentages aggregated by oxidation state, modification, strand, and coordinate. The matched unmethylated controls provide strand- and position-specific estimates of false-positive modification calls. The profiles are reported as measured modification percentages.

### 4.6. Data Analysis

The experimental design comprised four promoter variants, two oxidation states, and Dnmt3a-CD-treated and untreated conditions. One reaction was performed per experimental condition, with no independent replicate reactions. Variation across variants, strands, CpGs, or reads describes the analyzed preparations and does not estimate between-reaction variability.

The promoter-insert dataset contains 864 barcode × CpG × strand records for 27 CpGs (Table S4). The existing model-substrate plots and their aggregate summaries use these sites plus the flanking reference position at coordinate 3, giving 896 records. Restricting treated samples to valid coverage ≥20 gives 443 records in this 28-position set, or 429 within the 27-CpG insert. The flanking position is identified separately from the promoter CpGs. All textual CpG identifiers use the insert numbering, which also applies to the shared Raji sites.

For each barcode and strand, a weighted 5mC percentage was calculated from the modified-call count divided by valid coverage, summed across the selected positions. Overall variant values were obtained by averaging the C- and G-strand weighted percentages, followed by calculation of the mean and sample SD across the four variants. Mean C/G ratios were calculated from the four variant-specific ratios. These are descriptive summaries of different variants. G4Hunter [28,29] provided sequence-propensity scores, and PQS regions were assigned using the shared coordinates. Sequence-context comparisons used the template-corrected 9-mer column, including the 32 corrected rows whose windows overlap a substitution, with normalization within each barcode-and-strand group. Analyses emphasize methylation levels, profile correlations, and differences between the measured conditions. Figure 2C retains descriptive site-level regression fits and their confidence bands.

### 4.7. Bisulfite Sequencing

Raji genomic DNA was selected as a standardized human genomic substrate. The Dnmt3a-CD-treated aliquot was incubated with the enzyme for 1 h at 37 °C, converted with the BisQuick kit (Evrogen, Moscow, Russia), and amplified using 5′-GGGGTTAGGTYGGGTTTTTA-3′ and 5′-AAACCAAAACTTCCCACRTA-3′ primers. The 286-bp PCR product was sequenced on MinION using SQK-NBD114.24 (barcode24). Reads were aligned to the C–T-converted *hTERT* reference (chr5:1,292,808–1,297,757; NC_000005.10), and per-site estimates were calculated as 100 × C/(C + T) using samtools mpileup. Table S3 reports the 27 CpGs shared with the promoter insert, with approximately 9600–9900 informative calls per site. Per-position base qualities were also summarized by called base. The C-rich strand of an aliquot of the same Raji DNA was also analyzed without in vitro Dnmt3a-CD methylation, using the same bisulfite-conversion and PCR conditions (Figure S2; Table S5).

## 5. Conclusions

Dnmt3a-CD produced a strand-asymmetric de novo methylation pattern on the *hTERT* core promoter that was more even across strands and smoother within defined PQS cores, reduced in measured level but broadly retained after photo-oxidation, and largely robust to the clinically relevant C228T/C250T mutations. The same G-rich regions showed elevated background modification calls, which a quality threshold partly reduced. In Raji DNA, bisulfite analysis of treated and untreated aliquots identified a PQS-associated hypomethylation trough on the C-rich strand. No additional methylation attributable to Dnmt3a-CD was detected in this comparison. Together, these results support an association between defined PQS and the methylation pattern of the *hTERT* promoter in the analyzed model substrates and Raji DNA. They also highlight the difficulty of sequencing oxidation-prone, guanine-rich DNA, both for basic studies and for the clinical interpretation of *hTERT*-promoter methylation.

## Data Availability

The sequencing reads generated in this study have been submitted to the NCBI BioProject database https://www.ncbi.nlm.nih.gov/bioproject/ (accessed on 31 July 2026) under accession number PRJNA1506699.

## Supplementary Materials

The following supporting information can be downloaded at: https://www.mdpi.com/article/10.3390/ijms27209129/s1.

## Abbreviations

The following abbreviations are used in this manuscript:

5hmC	5-hydroxymethylcytosine
5mC	5-methylcytosine
8-oxoG	8-oxo-7,8-dihydro-2′-deoxyguanosine
AdoMet	S-adenosyl-L-methionine
CpG	cytosine–phosphate–guanine dinucleotide
Dnmt3a-CD	catalytic domain of DNA methyltransferase 3a
DNMT3A/DNMT3B	DNA methyltransferase 3A/3B
EDTA	ethylenediaminetetraacetic acid
ETS	E26 transformation-specific (transcription factor family)
G4	G-quadruplex
GABP	GA-binding protein
HEPES	4-(2-hydroxyethyl)-1-piperazineethanesulfonic acid
*hTERT*	human telomerase reverse transcriptase
PCR	polymerase chain reaction
PQS	putative quadruplex-forming sequence
Q-score	Phred quality score
SD	standard deviation
SDS-PAGE	sodium dodecyl sulfate polyacrylamide gel electrophoresis
WT	wild type
