# Supplementary material for: Single-Molecule Nanopore Sequencing of the TERT Core Promoter CpG Island Reveals PQS-Associated Strand Asymmetry of De Novo Methylation and Its Modulation by Photo-Oxidation
Source: Int J Mol Sci. 2026 Oct 15;27(20):9129. doi: 10.3390/ijms27209129 (PMC13705005; doi:10.3390/ijms27209129)
Supplement: Supplementary file 1 [file ijms-27-09129-s001.zip › Figure S1.docx]

**Figure S1.** **CpG map of the hTERT core-promoter amplicon**. Nucleotide sequence of the amplicon (cytosine-rich reference strand, shown 5′→3′) with the analyzed CpG dinucleotides marked and numbered 1–26 below their positions in 5′→3′ order.
