# Supplementary material for: Single-Molecule Nanopore Sequencing of the TERT Core Promoter CpG Island Reveals PQS-Associated Strand Asymmetry of De Novo Methylation and Its Modulation by Photo-Oxidation
Source: Int J Mol Sci. 2026 Oct 15;27(20):9129. doi: 10.3390/ijms27209129 (PMC13705005; doi:10.3390/ijms27209129)
Supplement: Supplementary file 1 [file ijms-27-09129-s001.zip › Figure S2.docx]

**Figure S2.** PQS-associated hypomethylation in Raji genomic DNA without in vitro Dnmt3a-CD treatment. Per-CpG bisulfite methylation of the C-rich C(+) strand is plotted along the amplicon coordinate frame used in Figure 5. Blue symbols show methylation values, and grey shading marks PQS3, PQS2 and PQS1 (left to right). A single aliquot of the same genomic DNA preparation used for Figure 5 was analyzed using the same bisulfite-conversion and PCR conditions.
